# Annual trends in heterozygosity of Korea native cattle (Hanwoo) based on microsatellite markers

**DOI:** 10.5713/ab.250594

**Published:** 2026-03-11

**Authors:** Eunho Kim, Changgwon Dang, Jaebeom Cha, Hyukkee Chang, Haseung Seong, Sangmin Lee, Woncheoul Park, Jongan Lee, Haesu Ko, Mahboob Alam, Dongkyu Lee, Eunah Ryu, Chaeyoung Lee, Ryunha Kim, Wooyoung Jung, Mina Park

**Affiliations:** 1 Animal Genetics & Breeding Division, National Institute of Animal Science, Cheonan, Korea; 2 Hanwoo Improvement Center for National Agricultural Cooperative Federation (NACF), Seosan, Korea

**Keywords:** Hanwoo, Heterozygosity, Inbreeding, Microsatellite Marker

## Abstract

**Objective:**

High-qulity Hanwoo (Korean native cattle) semen yields calves with better genetics, significantly enhancing farm profits. However, the repeated use of this semen can reduce heterozygosity and genetic diversity in the Hanwoo population, potentially compromising parentage verification accuracy. This study was conducted to analyze large-scale microsatellite (MS) marker data to evaluate the heterozygosity of Hanwoo cows and the discriminatory power of the MS marker set currently used for parentage verification.

**Methods:**

The study involved Hanwoo cows from farms participating in the Hanwoo cow improvement project, utilizing MS marker data from 778,544 heads collected for parentage verification since 2012. Heterozygosity (H_Obs_), expected heterozygosity (H_Exp_), polymorphism information content (PIC), and the inbreeding coefficient within populations (F_IS_) were estimated using R version 4.3.3 and Cervus version 3.0.7.

**Results:**

The results showed average values of H_Obs_, H_Exp_, and PIC were 0.771, 0.768, and 0.736, respectively. Heterozygosity by marker suggested a gradual decrease in variability for most markers post-2010. After 2010, the analysis of over 10,000 animals led to a decrease in variance of sample statistics, improving the accuracy of estimates. The F_IS_ values suggest that the population is approaching Hardy-Weinberg equilibrium and that inbreeding risk is being effectively managed through planned breeding programs. To assess trends in genetic differentiation over time, we grouped individuals by birth year (2001–23) and calculated pairwise genetic differentiation values. The values ranged from 0.0003 to 0.0081, indicating low genetic differentiation and suggesting temporal genetic stability.

**Conclusion:**

This study shows that the Hanwoo population has high genetic diversity and low fixation, and that the current MS marker set remains reliable for future parentage verification.

## INTRODUCTION

The Hanwoo, a native cattle breed in Korea, is economically, nutritionally, and culturally important in Korea. Known for its docile temperament, adaptability to temperature variations, and high productivity, the Hanwoo has systematically improved owing to national institutions implemented in the 1960s. The genotype of the Hanwoo has been improved by selecting Korean proven bull numbers (KPNs), which require an accurate construction of pedigrees. Although the current Hanwoo traceability system employs unique identification numbers to manage the information of individuals, it cannot verify an individual’s identification or perform parentage verification. To overcome this limitation, microsatellite (MS) markers have been utilized to verify parentage [1–3]. Animals develop unique genotypes through meiosis during gametogenesis, and MS markers offer a powerful tool for identifying individuals by detecting differences in repetitive base sequences [4,5]. This technique has facilitated the development of species-specific identification marker sets for individuals, both domestically and internationally. In Korea, a system of identifying individuals and verifying parentage was established using MS markers proposed by the International Committee for Animal Recording (ICAR) and the International Society for Animal Genetics (ISAG) [6,7]. However, with the availability of high-quality reference genomes and commercial SNP arrays for Hanwoo cattle, SNP-based parentage verification has become internationally recognized as a more accurate and standardized approach, as recommended by ICAR and ISAG [8]. In line with this shift, recent studies have highlighted the expanding use of genome-wide data in livestock improvement, including the incorporation of imputed SNP genotypes into genomic-polygenic evaluations and the use of whole-genome resequencing [9,10]. However, in Korea, routine SNP-based testing remains limited because most cattle farms have restricted access to SNP genotyping services. Due to its lower cost and faster turnaround time, MS analysis continues to be the most practical and cost-effective approach for parentage verification in Hanwoo cattle.

Through this technique, Hanwoo pedigree information could be adequately managed. This improvement in pedigree management has greatly increased the accuracy of genetic evaluations of the Hanwoo, resulting in a considerable preference for the KPNs of bulls that have semen with high genetic merit. Because calves produced from semen with superior genetic potential directly increase the income derived from farming, farmers strongly prefer semen from bulls with highly ranking KPNs. As a result, the carcass performance of steers, which are the primary animals used for beef production, has improved markedly over the past decade. The average carcass weight increased from 425 kg in 2014 to 475.8 kg in 2024, and the mean marbling score improved from 5.5 to 6.6 point during the same period [11,12]. These results demonstrate that continuous genetic improvement has been achieved in the Hanwoo population. However, previous studies have reported that an increased preference for and repeated use of specific KPNs can lead to a reduction in effective population size, increased genetic drift, and limited genetic improvement, potentially resulting in population extinction in extreme cases [13,14].

To prevent such negative effects, regular monitoring of the genetic diversity in Hanwoo populations is crucial. MS markers are widely used not only for individual identification and parentage verification but also to study genetic diversity [15–18]. Currently employed MS marker sets can the track temporal changes in the genetic diversity and heterozygosity of the Hanwoo. Therefore, this study analyzed MS marker information collected through the Hanwoo cow improvement project since 2012, aiming to identify temporal trends in the genetic diversity and heterozygosity of the Hanwoo population.

## MATERIALS AND METHODS

### Animals and microsatellite marker information

The test population used in the analysis consists of a group from the Hanwoo cow improvement project, which was initiated to promote cow improvement in the farms through pedigree management, growth, genomic analysis and genetic evaluation of Hanwoo cows. For the analysis, we used MS marker information from 969,597 heads collected for parentage verification from 2013 to 2023. To enhance the reliability of pedigree, parentage verification using MS markers has been routinely implemented in the Hanwoo breeding program since 2012. Pedigree inconsistencies were corrected based on MS genotyping results, and only animals with confirmed and error-free parentage information (n = 969,597) were retained for the present analysis. The MS markers used in the analysis include 11 dinucleotide repeat allele markers currently being used in the Hanwoo traceability system and 2 sex determination markers. These markers were selected based on national traceability standards and have been consistently applied in large-scale parentage testing. The MS marker panel is also aligned with the recommendations of the Food and Agriculture Organization of the United Nations (FAO) and the ISAG–FAO Advisory Group, which proposed 30 standard MS markers for genetic diversity studies in major livestock species, including cattle [19].

### Data preprocessing

The test population consisted of MS marker information from 969,597 heads (i). The data preprocessing was conducted in the following order (Table 1). Removed: 39,517 heads with missing allele values (ii), 377 heads with outlier allele values beyond the Genetrack ver. 2 range (iii), and 114,982 heads with sex determination marker anomalies (individuals with values other than X or Y, those with missing values, and individuals whose sex marker information did not match their registered sex in the Korea Animal Improvement Association database) (iv). 2,229 heads were added from duplicated parentage verification where analysis was possible (v). And 147 heads were removed from birth year groups containing fewer than 100 heads (vi). Finally, 778,544 heads (i–ii–iii–iv+v–vi) were selected for the analysis. Table 2 shows the distribution of the analyzed population by birth year. The data preprocessing procedure was performed using R [20].

### Statistical analysis

Basic analyses including allele frequency, H_Obs_, H_Exp_, and PIC of MS markers were performed using Cervus ver. 3.0.7 [21]. Subsequently, the data used in the Cervus analysis was converted to Genepop ver. 4.7.3 [22] format, and F_IS_, F_ST_ [23,24], was estimated using the hierfstat package in R.


(1) $$H_{Obs}=\frac{No.\;of\;heterozygous}{n}$$

(2) $$H_{Exp}=1-\sum_{i=1}^{n}p_{i}^{2}$$

(3) $$\text{PIC}=1-\sum_{i=1}^{n}p_{i}^{2}-\sum_{i=1}^{n-1}\sum_{j=t+1}^{n}2p_{i}^{2}p_{j}^{2}$$

Here, H_Obs_ is the observed heterozygosity and H_Exp_ is the expected heterozygosity, where n is number of alleles and pi is the frequency of the i^th^ alleles. PIC is the polymorphic information content, where n is the number of alleles, pi is the frequency of the i^th^ alleles, pj is the frequency of the j^th^ alleles.


(4) $$F_{IS}=\frac{H_{Exp}-H_{Obs}}{H_{Exp}}$$

(5) $$\text{pairwise }{\text{F}}_{ST}=\theta^{i,j}=\frac{H_{T}^{i,j}-H_{s}^{i,j}}{H_{T}^{i,j}}$$

F_IS_ is the inbreeding coefficient within populations. Pairwise F_ST_ indicates the genetic differentiation between two populations and is estimated using the *θ^i,j^* statistic as proposed by Weir and Cockerham [24]. $\theta =\frac{a}{a+b+c}$, a is between population component, b is within population component, c represents the sampling error. ${\text{H}}_{T}^{i,j}$ refers to the expected total heterozygosity across populations *i* and *j*, and ${\text{H}}_{S}^{i,j}$ is the average expected heterozygosity within those two populations. Non-exclusion probabilities (NE-1P, NE-2P, NE-PP, NE-I, and NE-SI) were calculated based on allele frequencies from the entire population using Cervus ver. 3.0.7 [21]. The identification accuracy for each index was derived as 1–NE, representing the probability of correctly identifying or excluding individuals.

## RESULTS

Table 3 presents the basic results obtained by analyzing the results from the tested population, including the number of alleles per MS marker (k), as well as the H_Obs_, H_Exp_, PIC, and F_IS_ values. A high level of polymorphism was observed across all analyzed markers, with an average number of alleles of 15.636 per locus (k). The *TGLA122* marker showed the highest number of alleles, with 28 alleles, whereas the *TGLA126* marker exhibited the lowest, with 9 alleles. The mean H_Obs_ and H_Exp_ values were 0.771 and 0.768, respectively. The *TGLA53* marker showed the highest level of heterozygosity, at 0.891 and 0.890, respectively, whereas the *TGLA126* marker showed the lowest heterozygosity level, at 0.669 and 0.665, respectively. Meanwhile, the average PIC value was 0.736, with *TGLA53* and *TGLA126* showing the highest and lowest values, respectively, which is consistent with the H_Obs_ and H_Exp_ results. The F_IS_ value, indicating the inbreeding coefficient of a population, had a mean of −0.003, and negative values were observed across all markers. *TGLA53* exhibited the highest value (−0.0014), whereas *TGLA126* showed the lowest value (−0.0053).

The results obtained by using 11 MS markers to calculate probabilities for individual identification and parentage verification are presented in Table 4. NE-1P, NE-2P, NE-PP, NE-I, and NE-SI represented the combined non-exclusion probabilities of the first parent, second parent, parent pair, identity, and sibling identity, respectively. The reliability of individual identification and parentage verification increased as these values approached zero. Furthermore, analyses of the performance of individual markers revealed that the *TGLA53* marker had the lowest error probabilities across all evaluation indices, indicating that it was the most effective marker for identification. Its error probabilities for NE-1P, NE-2P, NE-PP, NE-I, and NE-SI were 0.366, 0.223, 0.078, 0.022, and 0.311, respectively. Meanwhile, the *TGLA126* marker exhibited relatively high error probabilities across most indices.

An analysis combining all MS markers revealed high reliability for both individual identification and parentage verification. Non-exclusion probabilities (NE values) close to zero indicate higher exclusion power, meaning that unrelated or incorrect individuals are more likely to be successfully excluded. Accordingly, identification accuracy, calculated as 1–NE, approaches 1 when the marker set is highly discriminative. In this study, identification accuracies were 0.996914 (error probability: 0.003086), 0.9999349 (0.0000651), 0.99999991 (0.00000009), and 0.9999716 (0.0000284) for NE-1P, NE-2P, NE-PP, and NE-SI, respectively. Notably, the power for individual identification showed exceptionally high accuracy, at 0.99999999999924 (error probability: 0.00000000000076). These results suggest that the MS marker set used in this study was highly robust and reliable for both individual identification and parentage verification.

**Figure 1:**
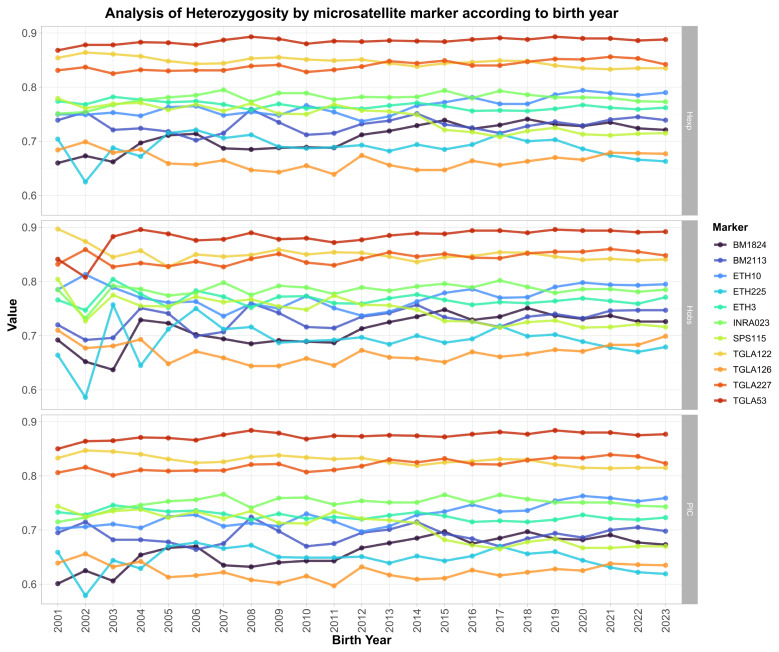
Annual changes in genetic diversity parameters (expected heterozygosity, H_Exp_; observed heterozygosity, H_Obs_; polymorphic information content, PIC) of Hanwoo cattle by birth year (2001–2023) based on 13 microsatellite markers. Each line represents the value of a specific marker across birth years. The three panels show (top) H_Exp_, (middle) H_Obs_, and (bottom) PIC, respectively. Symbols and colors correspond to different microsatellite loci as indicated in the legend.

Figure 1 illustrates the temporal changes in H_Obs_, H_Exp_, and PIC values of the MS markers according to birth year from 2001 to 2023. Overall, high levels of heterozygosity were maintained across all markers, with most values ranging between 0.6 and 0.9. During the early period (2001–2007), several markers, including BM1824, BM2113, and ETH225, exhibited notable year-to-year fluctuations, with ETH225 showing the most pronounced variation. This instability is likely attributable to the relatively small number of genotyped animals during those years (Table 1), which may have amplified annual variation. From 2008 to 2015, heterozygosity values across most markers became more consistent, and after 2015 they remained largely stable, indicating a gradual stabilization of genetic diversity within the Hanwoo population. Among the 11 markers analyzed, TGLA122, TGLA227, and TGLA53 consistently exhibited the highest heterozygosity (H_Obs_ and H_Exp_: 0.85–0.90), reflecting strong allelic diversity at these loci. In contrast, TGLA126 displayed the lowest heterozygosity (0.65–0.70), although it consistently remained above 0.6 throughout the study period, showing only minor temporal variation. BM1824 and ETH10 showed slight increases in heterozygosity over time, whereas INRA023 exhibited a modest decline after 2015 but still maintained a relatively high level overall. Taken together, these findings indicate that the genetic diversity of the Hanwoo population experienced moderate fluctuations in the early 2000s—largely due to smaller sample sizes—but has remained stable since 2008, showing no significant long-term loss of diversity between 2001 and 2023.

To assess temporal trends in genetic differentiation (F_ST_), we divided the population by birth year from 2001 to 2023 and compared them across years. The results are presented in Table 5. As shown in Table 5, the pairwise F_ST_ values ranged from 0.0003 to 0.0081, indicating an overall low level of genetic differentiation between birth years. The highest F_ST_ value (0.0081) was observed between the 2002 and 2023 birth years, while the lowest F_ST_ value (0) occurred between 2001 and 2002. These findings suggest that the genetic structure of the Hanwoo population has remained generally stable, likely due to consistent nationwide breeding programs. Nevertheless, F_ST_ values tended to increase slightly as the interval between birth years widened, which may reflect the cumulative effects of genetic drift or selection over generations.

**Figure 2:**
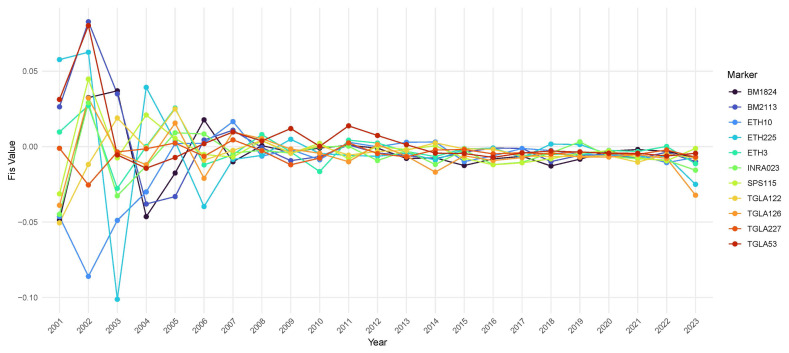
Annual changes in inbreeding coefficient (F_IS_) of Hanwoo cattle from 2001 to 2023 based on 13 microsatellite markers. Each colored line represents the F_IS_ value estimated for a specific marker in a given birth year. Positive values indicate an excess of homozygosity, whereas negative values indicate an excess of heterozygosity relative to Hardy–Weinberg equilibrium.

Changes in the F_IS_ values of the 11 MS markers for the period of 2001 to 2023 were analyzed and are presented Figure 2. Most markers tended to maintain F_IS_ values close to zero, suggesting that the tested population approached Hardy–Weinberg equilibrium. While significant variations existed among some markers during this period, the values gradually stabilized over time. The *ETH225* marker exhibited the lowest F_IS_ value (−0.1012) in 2003, but this value subsequently rapidly approached zero, reaching levels similar to those of the other markers. After 2005, most markers’ F_IS_ values fluctuated within the narrow range of −0.02 to 0.02. The *BM2113* and *TGLA53* markers initially showed positive values, which generally shifted toward slightly negative values closer to zero over time. Toward 2023, most markers exhibited negative F_IS_ values, with the *ETH225* and *TGLA126* markers showing highly negative values.

## DISCUSSION

The H_Obs_, H_Exp_, and PIC values by marker obtained in this study indicate that the tested population maintained stable genetic diversity (Table 3). The H_Obs_ and H_Exp_ values were similar across markers (>0.6), indicating high genetic diversity within the population. Previous studies on Hanwoo cattle showed results similar to those in this study. Specifically, Jin et al [25] reported H_Obs_, H_Exp_, and PIC values of 0.760, 0.757, and 0.722, respectively, after using 11 MS markers on Hanwoo populations, while Shin et al [26] reported values of 0.773, 0.764, and 0.727, respectively, for KPNs. The H_Exp_ values obtained in the present study met the criterion of 0.3–0.8 proposed by Takezaki and Nei [27], suggesting that the markers were suitable for diversity analysis. Regarding the PIC values used to assess genetic diversity, markers are considered highly polymorphic and most suitable when the PIC value exceeds 0.50, moderately suitable when the PIC value ranges between 0.25 and 0.50, and uninformative when the PIC value is less than 0.25 [28,29]. These criteria are commonly used when establishing MS marker sets for various livestock species [1,4,30]. Furthermore, analysis of marker discriminatory power revealed a notable difference among the indices (Table 4). The relatively higher error probability observed for NE-1P (0.003086) compared to other indices (NE-2P, NE-PP, NE-I, NE-SI) can be attributed to the inherent limitation of using only one parent’s genotype in parentage verification. While NE-PP utilizes both parental genotypes, NE-1P is based solely on either the sire or the dam, thereby increasing the likelihood that a non-parent may not be excluded. In previous studies, Weng et al [31] and Kim et al [32] reported NE-1P values of 0.0289 and 0.02464, respectively—both substantially higher than the value obtained in this study (0.003086). Moreover, the NE-2P, NE-PP, NE-I, and NE-SI values in this study were also lower than those reported in previous studies [33–35], further demonstrating the superior reliability and discriminatory capacity of the selected MS marker set. This exceptionally low NE-PP value (0.00000009) underscores the importance of obtaining complete parental genotypes to minimize error and improve parentage verification accuracy. Based on the findings, the MS marker set currently used in the system of identifying Hanwoo individuals and verifying their parentage is appropriate and reliable for analyzing Hanwoo genetics.

A notable result of the heterozygosity level by birth year was that most markers maintained a high heterozygosity level, without significant decreases over time (Figure 1). Generally, a high level of heterozygosity indicates a low inbreeding risk within a population. Meanwhile, the F_IS_ value ranges from −1 to 1, where higher positive values indicate more inbreeding and lower genetic diversity, whereas lower negative values indicate excessive heterozygosity due to artificial insemination and planned mating performed to avoid inbreeding. Values closer to zero indicate closer proximity to Hardy–Weinberg equilibrium, at which point allele frequencies remain stable over time [23]. This study obtained slightly negative values close to zero for all markers (Figure 2), similar to the findings reported in previous studies conducted on European, Asian, and African cattle breeds [36–39]. These results suggest that effective breeding management can be achieved through planned mating combinations, while avoiding inbreeding risks. And to assess temporal trends in genetic differentiation, we divided the population by birth year from 2001 to 2023 and compared them across years (Table 5). F_ST_ values range from 0 to 1, where values closer to 0 indicate little genetic differentiation, and values closer to 1 indicate greater differentiation [24,40]. The resulting values ranged from 0.0003 to 0.0081, indicating a generally low level of genetic differentiation among population across years. This suggests that the genetic structure of the population has remained largely stable over time. However, F_ST_ values tended to increase slightly as the interval between birth years widened, implying a gradual accumulation of genetic differentiation across generations [41]. Nonetheless, the overall F_ST_ values were very low, demonstrating that ongoing pedigree management and selection strategies have been effective in maintaining genetic diversity.

After 2010, the level of heterozygosity for all markers and populations varied minimally, maintaining a stable state (Figure 1, Table 5), which demonstrated the importance of adequate pedigree management and of farmers’ awareness of the risks associated with inbreeding. The number of animals analyzed increased significantly over time (Table 2). Before 2005, fewer than 1,000 animals were analyzed annually, but since 2010, this number increased to more than 10,000 annually. This substantial increase in sample size may be directly linked to reductions in heterozygosity variability (Figure 2). The Hanwoo cow improvement project, initiated to improve cows on farms, conducts parentage verification for 17,300 animals annually and, since 2012, has continuously managed information by verifying pedigrees and managing parentage verification results by using an MS marker set [12,42]. In large samples, according to statistical principles, as the variance of sample statistics decreases and the accuracy of estimates improves, estimates of the heterozygosity level across years become increasingly similar. From 2001 to 2009, the heterozygosity and F_IS_ values varied considerably, but this variability decreased significantly after 2010. This suggested that, as the size of the analyzed population increased, the statistical power and reliability of the estimates improved.

Continuous consulting by institutions that work toward improving Hanwoo populations, as well as the provision of mating plan guidelines, have increased the importance of parentage verification, leading to a higher number of animals being analyzed. As a result, a high level of heterozygosity and low level of genetic fixation have been observed, as shown in this study. The stability and temporarily increasing trend of the heterozygosity level observed for most markers after 2010 were considered results of adequate pedigree management achieved by emphasizing the importance of parentage verification, as well as by continuously monitoring pedigrees and implementing planned mating programs. The development of a mating plan is essential for maintaining both a high level of genetic diversity and low inbreeding rates and for establishing long-term improvement plans [43]. The results of this study demonstrated that improvements in cows have been achieved not only by enhancing productivity but also by maintaining genetic diversity, and the findings are anticipated to serve as important reference data for establishing future breeding programs or conservation strategies.

## CONCLUSION

The construction of accurate pedigrees for the Hanwoo has improved the accuracy of genetic evaluations, resulting in a preference for semen from highly ranked proven bulls, as calves produced from semen with superior genetic potential directly increase farm profitability. However, excessive use of specific KPNs may cause reductions in effective population sizes, along with genetic drift and limited genetic improvement, which may result in a decrease in genetic diversity in the long term. This study analyzed MS marker information collected through the Hanwoo cow improvement project since 2012, aimed at identifying trends in the genetic diversity and heterozygosity of the Hanwoo population. The average H_Obs_, H_Exp_, and PIC values were 0.771, 0.768, and 0.736, respectively, with H_Obs_ and H_Exp_ being highly similar across all markers, and the PIC values exceeding 0.6. An examination of the heterozygosity level of markers according to birth year revealed that, since 2008, the variability gradually decreased for most markers. After 2010, as the number of animals analyzed increased to over 10,000, the variance in the sample statistics decreased, indicating an improvement in the accuracy of estimates. The heterozygosity and F_IS_ values varied considerably from 2001 to 2009 but stabilized or increased after 2010. Additionally, toward 2023, the F_IS_ values generally showed negative values closer to 0. To assess temporal trends in genetic differentiation, we divided the population by birth year from 2001 to 2023 and compared them across years. the pairwise F_ST_ values ranged from 0.0003 to 0.0081, indicating an overall low level of genetic differentiation between birth years.

Based on the results of this study, it is concluded that the maintenance of low genetic fixation and high genetic diversity has been achieved through continuous parentage verification and through planned mating programs implemented by the Ministry of Agriculture, Food and Rural Affairs via the Hanwoo cow improvement project. The MS marker set that has long been used is expected to remain sufficiently reliable for future analyses of individual identification and parentage verification performed for the Hanwoo.

## Figures and Tables

**Table 1:** Pre-processing procedure and results of raw data for analysis

Type		Number of animal
Raw data		969,597 (i)
Missing allele value		39,517 (ii)
Allele value outlier		377 (iii)
Sex allele outlier	No X, Y value	29
	No has sex marker	91,275
	Mismatch sex marker	23,678
	Total	114,982 (iv)
Duplicate barcode ID QC		
Duplicate barcode ID	2 duplication	18,396
	3 duplication	489
Combining duplicate barcode ID for analysis data	2 duplication	2,226
	3 duplication	3
	Total	2,229 (v)
Remove if less than 100 heads		147 (vi)
Analysis data	A–B–C–D+E–F	778,544

**Table 2:** Number of animals used in analysis by birth year

Birth year	Frequency			

	Cow (head)	Bull (head)	Total (head)	Total (%)
2001	107	0	107	0.01
2002	198	0	198	0.03
2003	342	0	342	0.04
2004	546	1	547	0.07
2005	1,000	1	1,001	0.13
2006	1,672	0	1,672	0.21
2007	2,650	2	2,652	0.34
2008	4,227	2	4,229	0.54
2009	6,679	0	6,679	0.86
2010	11,719	0	11,719	1.50
2011	16,411	132	16,543	2.12
2012	24,500	1,088	25,588	3.29
2013	28,306	2,961	31,267	4.02
2014	33,680	4,777	38,457	4.94
2015	39,749	8,176	47,925	6.15
2016	48,430	12,682	61,112	7.85
2017	56,654	20,571	77,225	9.92
2018	59,580	31,503	91,083	11.70
2019	54,357	33,087	87,444	11.23
2020	57,964	47,420	105,384	13.53
2021	45,179	48,121	93,300	11.98
2022	33,098	38,635	71,733	9.21
2023	1,059	1,278	2,337	0.30
Total	528,107	250,437	778,544	99.97

**Table 3:** Basic analysis of microsatellite markers in test population

Locus	Type					

	k	N	H_Obs_	H_Exp_	PIC	F_IS_
*BM1824*	11	778,544	0.732	0.730	0.684	−0.0038
*BM2113*	17	778,544	0.736	0.733	0.692	−0.0034
*ETH10*	12	778,544	0.780	0.778	0.745	−0.0020
*ETH225*	11	778,544	0.693	0.691	0.649	−0.0022
*ETH3*	15	778,544	0.764	0.763	0.723	−0.0017
*INRA023*	16	778,544	0.788	0.784	0.755	−0.0040
*SPS115*	15	778,544	0.727	0.724	0.683	−0.0040
*TGLA122*	28	778,544	0.846	0.843	0.825	−0.0028
*TGLA126*	9	778,544	0.669	0.665	0.625	−0.0053
*TGLA227*	19	778,544	0.851	0.849	0.831	−0.0024
*TGLA53*	19	778,544	0.891	0.890	0.880	−0.0014
Mean	15.636	778,544	0.771	0.768	0.736	−0.0030

k, number of alleles; H_Obs_, observed heterozygosity; H_Exp_, expected heterozygosity; PIC, polymorphic information content; F_IS_, inbreeding coefficient.

**Table 4:** Analysis results of microsatellite markers identification power in test population

Locus	Type^1)^				

	NE-1P	NE-2P	NE-PP	NE-I	NE-SI
*BM1824*	0.681	0.507	0.324	0.118	0.415
*BM2113*	0.670	0.493	0.305	0.112	0.411
*ETH10*	0.608	0.429	0.245	0.082	0.381
*ETH225*	0.719	0.542	0.353	0.138	0.439
*ETH3*	0.641	0.463	0.283	0.096	0.393
*INRA023*	0.588	0.409	0.221	0.075	0.377
*SPS115*	0.679	0.502	0.314	0.117	0.417
*TGLA122*	0.479	0.311	0.140	0.043	0.339
*TGLA126*	0.741	0.563	0.372	0.152	0.455
*TGLA227*	0.467	0.302	0.133	0.041	0.336
*TGLA53*	0.366	0.223	0.078	0.022	0.311
All marker set mean	0.003086	6.51E-05	9.00E-08	7.60E-13	2.84E-05

1) NE-1P: Combined non-exclusion probability (first parent); NE-2P: Combined non-exclusion probability (second parent); NE-PP: Combined non-exclusion probability (parent pair); NE-I: Combined non-exclusion probability (identity); NE-SI: Combined non-exclusion probability (sib identity).

**Table 5:** Pairwise estimates of genetic differentiation (F_ST_) by birth year

Birth year	2001	2002	2003	2004	2005	2006	2007	2008	2009	2010	2011	2012	2013	2014	2015	2016	2017	2018	2019	2020	2021	2022	2023
2001		0.0000	0.0013	0.0016	0.0017	0.0022	0.0033	0.0027	0.0034	0.0030	0.0027	0.0037	0.0044	0.0057	0.0069	0.0065	0.0059	0.0065	0.0063	0.0060	0.0068	0.0072	0.0075
2002			0.0014	0.0007	0.0025	0.0047	0.0043	0.0044	0.0046	0.0031	0.0028	0.0033	0.0045	0.0068	0.0073	0.0069	0.0076	0.0073	0.0075	0.0069	0.0073	0.0079	0.0081
2003				0.0004	0.0013	0.0021	0.0029	0.0040	0.0034	0.0023	0.0017	0.0029	0.0039	0.0056	0.0060	0.0060	0.0063	0.0068	0.0069	0.0061	0.0069	0.0076	0.0080
2004					0.0010	0.0024	0.0022	0.0032	0.0028	0.0022	0.0014	0.0024	0.0034	0.0051	0.0053	0.0061	0.0057	0.0065	0.0068	0.0064	0.0071	0.0077	0.0081
2005						0.0007	0.0015	0.0027	0.0024	0.0018	0.0015	0.0019	0.0028	0.0043	0.0045	0.0046	0.0043	0.0051	0.0052	0.0051	0.0058	0.0065	0.0070
2006							0.0012	0.0022	0.0024	0.0021	0.0016	0.0022	0.0027	0.0036	0.0042	0.0043	0.0042	0.0052	0.0052	0.0051	0.0060	0.0066	0.0072
2007								0.0015	0.0019	0.0020	0.0018	0.0019	0.0025	0.0030	0.0034	0.0041	0.0039	0.0048	0.0048	0.0048	0.0055	0.0058	0.0064
2008									0.0015	0.0027	0.0025	0.0021	0.0022	0.0026	0.0035	0.0037	0.0042	0.0044	0.0042	0.0046	0.0052	0.0055	0.0060
2009										0.0010	0.0017	0.0021	0.0023	0.0028	0.0029	0.0033	0.0037	0.0044	0.0044	0.0040	0.0047	0.0050	0.0055
2010											0.0009	0.0021	0.0027	0.0037	0.0033	0.0031	0.0040	0.0047	0.0049	0.0040	0.0045	0.0050	0.0054
2011												0.0012	0.0018	0.0033	0.0038	0.0041	0.0047	0.0055	0.0056	0.0050	0.0057	0.0064	0.0071
2012													0.0006	0.0020	0.0028	0.0030	0.0034	0.0034	0.0036	0.0036	0.0039	0.0046	0.0052
2013														0.0010	0.0020	0.0025	0.0033	0.0031	0.0028	0.0027	0.0032	0.0039	0.0044
2014															0.0008	0.0020	0.0026	0.0025	0.0020	0.0020	0.0024	0.0029	0.0034
2015																0.0014	0.0023	0.0024	0.0022	0.0020	0.0022	0.0026	0.0031
2016																	0.0019	0.0020	0.0016	0.0013	0.0014	0.0016	0.0017
2017																		0.0007	0.0015	0.0018	0.0020	0.0021	0.0024
2018																			0.0006	0.0012	0.0012	0.0017	0.0021
2019																				0.0005	0.0009	0.0015	0.0019
2020																					0.0004	0.0010	0.0012
2021																						0.0003	0.0008
2022																							0.0003
2023																							

F_ST_ values range from 0 to 1, where values closer to 0 indicate little genetic differentiation, and values closer to 1 indicate greater differentiation.

## Data Availability

Upon reasonable request, the datasets of this study can be available from the corresponding author.
